# Effects of Online Supervised Slow-Movement Bodyweight Resistance Training Followed by Self-Directed Exercise on Physical Function in Older Adults: A Pilot Study

**DOI:** 10.3390/healthcare13233091

**Published:** 2025-11-27

**Authors:** Zhenyue Liu, Shuji Sawada, Hisashi Naito, Shuichi Machida

**Affiliations:** 1 Institute of Health and Sports Science & Medicine, Juntendo University, Chiba 270-1695, Japan; z.liu.uo@juntendo.ac.jp (Z.L.); hnaitou@juntendo.ac.jp (H.N.); 2 Graduate School of Health and Sports Science, Juntendo University, Chiba 270-1695, Japan; sh-sawada@juntendo.ac.jp

**Keywords:** remote intervention, home-based resistance training, behavioral change, physical fitness

## Abstract

**Background/Objectives**: The development of safe and effective online exercise interventions is essential for enhancing physical activity among older adults and supporting healthy aging. However, the effects of online slow-movement bodyweight resistance training (SBRT) programs and exercise continuation after such interventions remain unclear. This pilot study examined the effects of a seven-week online exercise class (OEC) using SBRT on physical function in community-dwelling older adults and further explored exercise continuation and its effects on physical function during a subsequent seven-week self-directed exercise (SDE) period. **Methods**: Eight community-dwelling older adults (age: 70.0 ± 7.7 years) participated in a seven-week OEC (twice per week), followed by a seven-week SDE. Their physical function was assessed remotely using the 30-second chair stand test (CS-30) at baseline, after the OEC, and following the SDE. Training frequencies and volumes were recorded throughout the periods to evaluate exercise adherence and continuation. **Results**: The participants’ CS-30 performance significantly improved after the OEC (21.3 ± 2.7 vs. 24.0 ± 3.2 times; *p* = 0.004) and even more following the SDE (24.0 ± 3.2 vs. 27.8 ± 3.8 times; *p* = 0.002). The training frequency during the SDE period was comparable to that during the OEC period (2.5 ± 1.2 vs. 2.1 ± 1.0 days/week; *p* = 0.916), with there being no significant difference in lower limbs training volume between the two periods (1313.1 ± 376.4 vs. 1421.3 ± 673.2 repetitions; *p* = 0.698). **Conclusions**: A seven-week SBRT-based OEC may be a feasible remote exercise intervention that effectively improves physical function in older adults and encourages post-intervention exercise continuation.

## 1. Introduction

Due to accelerated global population aging, healthy life expectancy extension has become a critical public health issue [1,2]. Increasing physical activity levels through regular exercise, particularly resistance training, benefits the maintenance and improvement of muscle function and serves as a key strategy to prevent sarcopenia, frailty, and locomotive syndrome [3,4,5]. However, older adults often face multiple barriers to initiating and maintaining regular exercise, including the absence of exercise facilities in their vicinity, limited transportation options [6,7], and their lack of awareness of physical activity’s benefits [7]. Furthermore, during the coronavirus disease 2019 pandemic, lockdown restrictions caused a substantial reduction in people’s physical activity levels [8], with particularly pronounced impacts on older adults [9], which exacerbated the risk and severity of sarcopenia and frailty [10,11].

In recent years, interest on home-based exercise training programs has been increasing [12,13]. Currently, the integration of information and communication technology (ICT) into exercise programs, enabling the provision of real-time instruction and feedback to help older adults receive adequate exercise guidance and improve exercise quality [14], is considered a feasible approach. Hence, the development of safe and effective ICT-based online exercise interventions is essential to promote exercise participation and enhance physical activity levels among older adults to support healthy aging.

To date, many studies have implemented online exercise interventions for older adults using two-way ICT tools such as Zoom, Skype, and Facebook. These interventions involve various exercises, including aerobic dance [15]; resistance training [16,17]; and multicomponent exercise programs combining resistance, balance, flexibility, and aerobic exercises [18,19,20,21,22,23]. These studies highlight such online exercise programs’ feasibility for older adults and clarify different exercise types’ effectiveness in improving specific aspects of physical fitness. Accordingly, online exercise interventions are considered promising alternatives to traditional face-to-face programs, particularly for older adults. However, earlier studies primarily focused on online exercise interventions’ effects during the intervention period itself, providing only limited information on whether older adults maintained their exercise habits after program completion, that is, whether the online interventions could promote behavioral changes.

Our previous studies indicate that a 12-week slow movement bodyweight-based resistance training (SBRT) program, comprising nine exercises, delivered either through a group-supervised exercise class or in combination with unsupervised home exercise effectively improves older adults’ muscle mass, strength, and physical function without any adverse events [24]. However, the feasibility and effectiveness of implementing this safe and effective SBRT protocol in an online exercise setting for older adults remain unclear. 

Meanwhile, the 30-second chair stand test (CS-30), a physical function assessment for lower limb muscle strength evaluation [25], is as a useful tool for sarcopenia risk assessment in older adults [26]. Since CS-30 does not require any specialized equipment, it is particularly suitable for home-based assessments, and research confirms its feasibility and reliability in older adults’ remote evaluation through Zoom [27]. Based on these findings, SBRT and CS-30 appear to be promising tools for developing online exercise interventions and outcome assessments.

Therefore, in this pilot study, an online exercise class (OEC) using SBRT was delivered through Zoom. Following the class, a self-directed exercise (SDE) period of equal duration was implemented to evaluate exercise continuation among older adults. Three remote assessments were conducted throughout the two periods, CS-30 being the primary measure of intervention effects. Unlike our previous offline group-based exercise classes, this pilot study’s OEC spanned seven weeks and involved a reduced number of exercise types (four) to enhance the feasibility of home-based implementation. The aims of this study are to evaluate the effects of a seven-week SBRT-based OEC on physical function in older adults and assess exercise continuation during the subsequent SDE period.

## 2. Materials and Methods

### 2.1. Participants

We recruited 10 participants across Japan through a website (https://juntendo-kinkatsu.com/). The inclusion criteria were as follows: community-dwelling older adults who had no history of cardiovascular disease, fracture within six months, or movement disorders; had not engaged in regular resistance training for at least one year; and were approved by a physician to participate in this study’s exercise program. We informed the participants in advance through telephone or e-mail of the need to prepare a communication device, such as a computer, tablet, or smartphone having a camera and audio, and clarified how to download and use the ICT tool Zoom (Version 5.7, Zoom Video Communication, San Jose, CA, USA). Subsequently, we conducted a pre-study briefing session through Zoom to inform the participants of the study’s methods, procedures, and risks; further, they provided written informed consent for participation by post. Among the 10 recruits, 1 did not meet the inclusion criteria due to resistance training habits before the intervention, and another’s data were insufficient because they did not provide measurement results and training recording sheets; accordingly, these two participants were excluded from the study. Finally, eight participants (five women and three men, mean age = 70.0 ± 7.7 years) were included in the study’s analyses. Due to the pilot study’s exploratory nature, we did not perform an a priori power analysis to determine sample size.

### 2.2. Study Design

The study’s eight participants attended the OEC twice weekly for seven weeks, and relevant measurements were conducted before (Measurement 1) and after (Measurement 2) the OEC period to evaluate the OEC intervention effect. Subsequently, a seven-week SDE period was implemented, and a third measurement (Measurement 3) was conducted after the SDE period. A brief summary of the CS-30 improvement observed during the SDE period has been previously reported as a Letter to the Editor (Aging Medicine and Healthcare, accepted October 2025) [28]. The amount of lower limb training performed during the OEC and SDE periods was surveyed separately to assess exercise continuation. All participants attended the exercise classes and assessments remotely from their homes via Zoom. Two-way interaction with the participants was maintained throughout the study via e-mail, telephone, and Zoom.

This study was conducted in accordance with the guidelines of the Declaration of Helsinki and approved by the Ethics Committee of the Juntendo University Graduate School of Health and Sports Science Institutional Review Board (Approval Number: 2021-50). This study was also registered in UMIN-Clinical Trial Registry (trial ID: UMIN000044140).

### 2.3. Online Exercise Class

#### 2.3.1. OEC Implementation Mode and Flow

The OEC was conducted twice weekly for seven weeks using Zoom. In the first week, participants were provided general guidance and practice to adapt themselves to the OEC. Further, the training program was initiated in the second week (third lesson). We arranged a regular meeting room in advance for the OEC and explained in detail to the participants how to enter this meeting room through a unique uniform resource locator (URL) code in the pre-study briefing session. In addition, we prerecorded video instructional materials for all the training items included in the training program and developed warm-up and cool-down exercises for instruction. 

On each lesson day, study staff confirmed attendance and followed up with absent participants via phone or e-mail. Before each session, a brief safety check was conducted in which participants counted their resting pulse under the instructor’s guidance and confirmed whether they felt well enough to exercise; this screening was used only to ensure readiness for participation and was not collected as study data. A prerecorded warm-up video was then played via Zoom screen sharing, and participants performed warm-up exercise accordingly. Before each training item, the instructor explained key points such as posture and movement rhythm. Participants then practiced two to three repetitions while the instructor monitored and corrected their form. Subsequently, the instructor played prerecorded videos through Zoom, and the participants performed the training while watching and following the rhythm of the training in instructional videos. To confirm that the participants were performing the exercise in the correct posture, we instructed them to turn on Zoom’s camera function during the exercise and examined whether their entire body image could be seen on the monitor. In addition, to confirm the accuracy of participants’ movement posture from different angles, they were asked to exercise facing the camera during the first training set, turn sideways in the second, and exercise in their preferred direction during the third set. A professional instructor assessed training progress on the monitor and provided advice on training status to the participants during their rest periods between training sets and items. After the training program, cool-down exercises were conducted in the same manner as the warm-up. Finally, a question–answer session and summary were conducted at the end of each lesson. 

The participants were instructed to record their daily training status (if any), such as the number of repetitions and rate of perceived exertion score at the end of each set, sets, and training name on a prescribed recording sheet throughout the seven-week OEC, and avoid resistance training external to the OEC. The recording sheets were mailed to the participants in advance with instructions to mail them back at the end of the OEC (after Measurement 3).

#### 2.3.2. Training Program

The exercise program comprised the following four types of bodyweight-based resistance training: squat (SQ), split squat (SSQ), shoulder press (SP), and chair-seated abdominal curl (CSAC). The program focused on lower limb training (SQ and SSQ, with SSQ being performed on each foot separately) to improve the physical function associated with lower limb muscle strength. In addition, the training program used the participant’s own bodyweight as a load, without requiring any special equipment. Moreover, to perform CSAC, all that the participants needed for the entire training program was a chair approximately 40 cm high. From a practical standpoint, unlike our previous 9-exercise SBRT protocol, the simplified 4-exercise program was intentionally selected because several exercises in the original program were not suitable for remote implementation. Some of the nine exercises required specific equipment or camera angles that made safe execution or accurate form monitoring difficult in an online environment. These design choices aimed to select movements that older adults could perform safely and consistently at home while maintaining a primary focus on lower-limb training. The training started with lesson 3, and the participants performed two sets of eight repetitions of SQ with a 60-second rest in between. They were instructed to slowly complete each concentric and eccentric phase of movement by spending 3 s for each phase. Subsequently, the numbers of training items, repetitions, and sets per training were gradually increased, and the rest interval between the sets was slowly reduced. The final program was implemented from the 13th lesson onward, and it eventually included all the four items SSQ, SQ, SP, and CSAC, with 15 repetitions, three sets, and 30-second rest between sets for each item. Table 1 presents the exercise program’s details.

### 2.4. Self-Directed Exercise (SDE)

The seven-week OEC was followed by a seven-week SDE, which was attended by all eight participants. The participants were not required to continue exercising and were free to maintain the exercise habits developed during the OEC. We uploaded video teaching materials for all the training sessions on the website (https://juntendo-kinkatsu.com/, accessed on 15 March 2025). Further, we informed the participants that they could log on to the website to watch videos as they trained, confirm exercises’ rhythm and movements, and ensure the SDE’s training quality. Participants were instructed to record their exercise status on prescribed recording sheets (same as that during the OEC). We mailed the recording sheets to each participant before the SDE, and regular contact was maintained via phone and e-mail throughout the period. At the end of the SDE period, all the participants were instructed to mail their completed recording sheets back to the university.

### 2.5. Measurements

#### 2.5.1. Physical Function

To assess participants’ lower limb strength–related physical function, we conducted CS-30, which was completed by the participants from home using Zoom. The CS-30 test result was counted as the number of times a participant could stand from the sitting posture on a 40 cm high seat without using their arms in 30 s. The test protocol and requirements were explained in detail during the briefing session.

During measurement, the participants were instructed to turn on Zoom’s camera function and adjust their position to ensure that the instructor could see them on the monitor and confirm their movements’ accuracy. Once all conditions were prepared, the test began; the instructor controlled the 30-second time and provided start and end prompts. All the participants completed the test simultaneously online from their own homes. The instructor used Zoom’s video recording function to record the measurement session, and two researchers confirmed the accuracy of the participants’ movements and the number of times they completed the test by reviewing the recorded video after the measurement session. McCain et al. (2023) [27] reported the validity of the online CS-30 test using Zoom among community-dwelling older adults with an intraclass correlation coefficient of 0.94.

#### 2.5.2. Questionnaires

The Kihon Checklist (KCL) and 25-question geriatric locomotive function scale (GLFS-25) questionnaires were used at each measurement to assess the daily living function. The KCL questionnaire comprises 25 items with a total score of 25 points [29]. Accordingly, the participants’ height and weight data were self-reported in KCL, question 12. Further, GLFS-25 comprises 25 items with a total score of 100 points [30]. Before each measurement session, the questionnaires were mailed to the participants’ homes and, after each session, they returned the completed questionnaires to the university.

#### 2.5.3. Training Recording Sheet to Assess Training Status

To assess their training status during OEC and SDE, participants were asked to record their daily exercise training on a prescribed recording sheet. The sheets were mailed to the participants before the OEC and SDE and collected after the exercises.

The main recording sheet items were the type of training, number of sets, and number of repetitions in each set. Since the training program in this study focused mainly on lower limb training, we counted the training repetitions of SQ and SSQ during the OEC and SDE periods. Additionally, we counted the number of days on which the participants performed training during the OEC (including class attendance days) and SDE periods. These indicators were included in the analysis as indicators of the participants’ OEC and SDE training.

### 2.6. Statistical Analysis

Data are presented in the mean ± standard deviation (SD) and median [interquartile range] forms. The data’s normal distribution was assessed using the Shapiro–Wilk test. Further, changes in CS-30, KCL, and GLFS-25 values during OEC and SDE (Measurements 1–3) were analyzed using one-way repeated measures analysis of variance (ANOVA) or Friedman’s test. If ANOVA yielded a significant main effect of time, a Bonferroni correction was performed for the post hoc test. The training statuses for OEC and SDE, including the frequency of training and number of repetitions of lower limb training, were compared using the independent samples *t*-test and Mann–Whitney U test. All statistical analyses were conducted using SPSS (Version 29.0, IBM, Tokyo, Japan) with a significance level of *p* < 0.05.

## 3. Results

All eight participants completed 14 weeks of study, including all three measurement sessions, and consistently submitted questionnaires and training status records during the OEC and SDE periods. The average OEC attendance rate was 98.2 ± 3.3%. Participants did not report any exercise-related injuries or adverse events throughout the study. They had the following baseline characteristics: mean height, 157.5 ± 10.5 cm; weight, 54.9 ± 11.7 kg; and body mass index, 21.9 ± 2.5 kg/m^2^.

**Figure 1:**
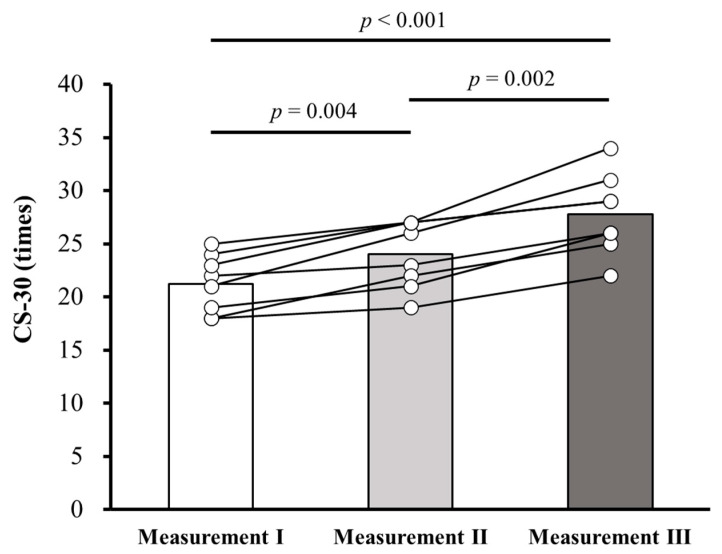
Changes in CS-30 performance during OEC and SDE. CS-30: 30-second chair stand test; OEC: online exercise class; SDE: self-directed exercise. Measurement 1: before the OEC period; Measurement 2: after the OEC or before the SDE period; Measurement 3: after the SDE period.

Table 2 presents changes in CS-30 times, total KCL scores, and total GLFS-25 scores over 14 weeks (Measurements 1–3). A significant main effect of time was observed for CS-30 (F = 50.38, *p* < 0.001, $\eta_{p}^{2}$ = 0.878), whereas no significant main effects of time were found for KCL (*p* = 0.112) or GLFS-25 (*p* = 0.542) scores. Post hoc analyses revealed significant improvements in CS-30 performance following both the OEC (Measurement 1 vs. Measurement 2; 21.3 ± 2.7 vs. 24.0 ± 3.2, mean difference: 2.75, 95% confidence interval (CI): 1.11–4.40, *p* = 0.004; Figure 1) and SDE (Measurement 2 vs. Measurement 3; 24.0 ± 3.2 vs. 27.8 ± 3.8, mean difference: 3.75, 95% CI: 1.81–5.69, *p* = 0.002; Figure 1) periods.

Table 3 presents the training statuses for OEC and SDE periods. There was no significant difference in weekly training frequency between the periods (2.5 ± 1.2 vs. 2.1 ± 1.0 days/week; *p* = 0.916). Similarly, no significant differences were observed in the number of repetitions performed between the two periods for SQ (528.4 ± 134.7 vs. 462.0 ± 242.8 repetitions; *p* = 0.510), SSQ (392.4 ± 135.5 vs. 479.6 ± 220.7 repetitions; *p* = 0.247) or total lower limb training volume (1313.1 ± 376.4 vs. 1421.3 ± 673.2 repetitions; *p* = 0.698).

## 4. Discussion

This pilot study evaluated the effects of a seven-week OEC using SBRT delivered through Zoom on older adults’ physical function. Additionally, we assessed how exercise continuation during a subsequent seven-week SDE period affected physical function. Results revealed significant improvement in CS-30 performance after OEC and further enhancements after the SDE period. Notably, participants maintained similar training volumes during the SDE and OEC periods.

Previous studies have revealed that a 12-week face-to-face SBRT program comprising nine exercises effectively improved CS-30 performance in older adults [24]. The current study’s findings are consistent with these results, although the OEC intervention was short (seven weeks) and incorporated only four SBRT exercises. Accordingly, even a brief and simplified SBRT program, when delivered remotely, can significantly enhance lower limb strength in older adults. In the current study, physical function was assessed using CS-30 alone without any direct measurements, such as dynamometers or ultrasound. However, earlier research indicates significant correlation of CS-30 performance with lower limb strength [26] and anterior thigh muscle thickness [31]. Hence, the intervention probably resulted in improvements in other aspects of muscle function, as well, warranting further investigation involving objective physiological assessments. Importantly, all aspects of this study—from recruitment to intervention delivery and assessments—were conducted remotely without any in-person contact. Even in this case, the OEC achieved a high adherence rate (98.2%), and no exercise-related adverse events occurred. This underscores the feasibility and safety of SBRT-based OEC as a completely remote exercise intervention for community-dwelling older adults. Moreover, these findings highlight this approach’s potential for broad dissemination and implementation.

Unlike the significant improvement observed in CS-30, no significant time effects were noted for KCL or GLFS-25 scores. A ceiling effect may partly explain these findings. In this study, participants had relatively healthy baseline status, with mean KCL and GLFS-25 scores of 5.0 and 9.8, respectively. The KCL is a frailty assessment tool with a commonly used cut-off score of ≥8 [29], and the GLFS-25 is a locomotive syndrome questionnaire with a cut-off score of ≥16 [30]. Thus, most participants were below the risk range at baseline, and the potential for improvement on these multidimensional scales was limited in our sample. Additionally, the present intervention primarily focused on improving physical function through a relatively short online exercise program followed by self-directed exercise. While this approach may effectively enhance physical function, its impact on the broader domains assessed by KCL and GLFS-25 (such as social participation, physical function, nutritional status, and psychological health) is likely to be limited. Therefore, future studies should consider longer intervention periods and more comprehensive, multifaceted programs that combine exercise with other strategies to influence these wider dimensions of health.

Exercise continuation during the SDE period was another important finding of this study. Participants maintained a comparable exercise frequency (median = 2.5 days/week) and training volume during SDE, resulting in continued improvements in CS-30 performance. Research reports that the benefits of short-term resistance training (4–8 weeks) can decline or even return to baseline after detraining periods [32,33]. Hence, the sustained improvements observed in such reports emphasize the importance of consistent exercise engagement in maintaining older adults’ physical function gains. Moreover, our results revealed significant correlations among changes in training volume and CS-30 performance, further reinforcing the critical role of ongoing exercise.

Exercise continuation was assessed based on the participants’ self-reported training logs and sustained exercise performance following OEC. Studies indicate that traditional machine-based resistance training programs do not always promote long-term adherence and motivation among older adults [34]. In contrast, the SBRT program implemented in this study used bodyweight alone as resistance and did not require any specialized equipment, which likely enhanced its feasibility for home-based training. Furthermore, the structured guidance, real-time feedback, and regular online assessments provided during the OEC period probably increased self-efficacy—a key determinant of exercise adherence [35,36]. These elements likely helped maintain motivation and supported transition to self-directed exercise behavior. However, since this study relied solely on training logs to assess exercise continuation and did not examine any underlying psychological factors, future research should incorporate wearable devices to facilitate objective monitoring and psychological assessments, such as self-efficacy scales that comprehensively examine the mechanisms promoting sustained exercise behavior.

Despite its promising findings, this study had several limitations. First, the participants were recruited through a website, suggesting that they may have had greater interest in health and higher digital literacy than the general older adult population. Together with the small sample size, this limits the generalizability of our findings. To avoid this limitation, future studies should recruit more diverse samples to enhance external validity. Second, this study used a single-arm design without a control group. Future studies should conduct randomized controlled trials to thoroughly evaluate the effects of the OEC and compare the effectiveness of OEC with that of traditional face-to-face exercise programs. Third, although this study included a seven-week SDE period to examine exercise continuation, it failed to clarify the long-term effects of OEC on exercise adherence and physical function. Finally, only the CS-30 was used to assess physical function, and the possibility of measurement error due to remote assessment cannot be excluded. To capture a more comprehensive picture of functional changes, future studies should incorporate a broader range of home-based assessments, such as balance or mobility tests.

## 5. Conclusions

According to this pilot study, SBRT-based online exercise classes delivered through Zoom may be a feasible and effective intervention to improve physical function in older adults. Moreover, such programs may promote the continuation of exercise behavior even after the structured intervention ends. These findings suggest that an intervention model combining an initial supervised online phase with a subsequent self-directed phase may represent a practical ICT-based remote health management strategy to support healthy aging.

## Figures and Tables

**Table 1:** OEC training program.

Lesson	Exercise	Repetition	CON-ECC	Set	Interval
3	SQ	8	3–3	2	60
4	SQ, SP	8	3–3	2	60
5	SQ, SP	10	3–3	2	60
6	SQ, SP, CSAC	10	3–3	2	60
7	SSQ, SQ, SP, CSAC	10	3–3	2	45
8	SSQ, SQ, SP, CSAC	10	3–3	3	45
9	SSQ, SQ, SP, CSAC	10	3–3	3	45
10	SSQ, SQ, SP, CSAC	15	3–3	3	45
11	SSQ, SQ, SP, CSAC	15	3–3	3	45
12	SSQ, SQ, SP, CSAC	15	3–3	3	30
13	SSQ, SQ, SP, CSAC	15	3–3	3	30
14	SSQ, SQ, SP, CSAC	15	3–3	3	30

OEC, online exercise class; SQ, squat; SP, shoulder press; CSAC, chair-seated abdominal curl; SSQ, split squat, conducted on each foot separately; CON-ECC, the time (in seconds) in each repetition’s concentric and eccentric phases; Interval, the rest time (in seconds) between the sets of each exercise.

**Table 2:** Changes in participants’ physical function and daily living function during OEC and SDE.

Outcome	Measurement 1	Measurement 2	Measurement 3	Main Effect of Time *
CS-30 (times)	21.3 ± 2.7 21.5 [18.3, 23.8]	24.0 ± 3.2 24.5 [21.3, 27.0]	27.8 ± 3.8 27.5 [25.3, 30.5]	*p* < 0.001
KCL (points)	5.0 ± 2.3 5.0 [2.5, 7.5]	3.6 ± 0.9 4.0 [3.0, 4.0]	3.9 ± 2.0 3.5 [3.0, 4.8]	*p* = 0.112
GLFS-25 (points)	9.8 ± 5.6 9.5 [6.3, 10.8]	8.8 ± 4.3 7.0 [6.3, 9.5]	9.8 ± 3.8 9.5 [7.3, 10.8]	*p* = 0.542

Data are shown as mean ± standard deviation and median [interquartile range]. * Data from CS-30 and KCL were analyzed by one-way repeated measures analysis of variance, and those from GLFS-25 were analyzed by Friedman’s test. CS-30, 30-second chair stand test; KCL, Kihon Checklist; GLFS-25, 25-question geriatric locomotive function scale.

**Table 3:** Comparison of Training Statuses for OEC and SDE Periods.

Outcome	OEC Period Measurements 1–2	SDE Period Measurements 2–3	*p*-Value
Days of training (days)	17.5 ± 8.7 14.0 [12.0, 22.5]	15.0 ± 6.9 17.5 [9.0, 19.8]	0.916 ^a^
Days of training/week	2.5 ± 1.2 2.0 [1.7, 3.2]	2.1 ± 1.0 2.5 [1.3, 2.9]	0.916 ^a^
Performed repetitions of SQ (reps)	528.4 ± 134.7 558.0 [382.5, 655.3]	462.0 ± 242.8 550.5 [270.0, 581.3]	0.510 ^b^
Performed repetitions of SSQ (one side) (reps)	392.4 ± 135.5 342.5 [305.0, 447.3]	479.6 ± 220.7 523.5 [315.0, 573.8]	0.247 ^a^
Performed repetitions of total lower limb training (reps)	1313.1 ± 376.4 1302.5 [992.5, 1520.0]	1421.3 ± 673.2 1552.5 [922.5, 1728.8]	0.698 ^b^

Data are shown as mean ± standard deviation and median [interquartile range]; OEC, online exercise class; SDE, self-discipline exercise; SQ, squat; SSQ, split squat; lower limb training, squat and split squat (two sides). ^a^, Mann–Whitney U test. ^b^, Independent samples *t*-test.

## Data Availability

The data that support the findings of this study are not publicly available due to privacy and ethical restrictions, but are available from the corresponding author upon reasonable request.

## Abbreviations

The following abbreviations are used in this manuscript:

OEC	Online exercise class
SBRT	Slow movement bodyweight-based resistance training
SDE	Self-directed exercise
CS-30	30-second chair stand test
ICT	Information and communication technology
SQ	Squat
SSQ	Split squat
SP	Shoulder press
CSAC	Chair-seated abdominal curl
KCL	Kihon Checklist
GLFS-25	25-question geriatric locomotive function scale
